# Metagenomic analysis reveals methanogenic and other archaeal genes in the digestive tract of invasive Japanese beetle larvae and associated soil

**DOI:** 10.3389/fmicb.2025.1609893

**Published:** 2025-07-25

**Authors:** Helena Avila-Arias, Michael E. Scharf, Ronald F. Turco, Diego J. Jiménez, Audrey Simard, Douglas S. Richmond

**Affiliations:** ^1^Department of Entomology, Purdue University, West Lafayette, IN, United States; ^2^Entomology and Nematology Department, University of Florida, Gainesville, FL, United States; ^3^Department of Agronomy, Purdue University, West Lafayette, IN, United States; ^4^Biological and Environmental Sciences and Engineering Division, King Abdullah University of Science and Technology, Thuwal, Saudi Arabia; ^5^Department of Entomology, Pennsylvania State University, University Park, PA, United States

**Keywords:** methane (CH_4_), Scarabaeidae, archaeome, midgut, hindgut

## Abstract

The linkage between methane emissions and the metabolic activity of archaeal species is broadly established. However, the structural and functional dynamics of this phenomenon within the scarab larval gut and associated host soil environment have not been investigated. In this study, we used shotgun metagenome sequencing to explore the archaeal communities associated with the digestive tract of third instar Japanese beetle (*Popillia japonica* Newman; Coleoptera: Scarabaeidae) (JB) larvae and its host soil. Our findings showed that both the JB gut compartment (midgut *vs*. hindgut) and experimental conditions (field *vs*. manipulative laboratory studies) significantly affect the composition of archaeal taxa. Moreover, gut compartment affected the functional profile. Results revealed an increase of methane metabolism-related taxa and gene sequences in the larval hindgut, supporting the hypothesis that methanogenesis is primarily maintained in that gut compartment. Methane production associated with the JB larval gut takes place primarily via CO_2_ reduction (~30%) and methanol methanation (4%) pathways. The presence of the same archaeal features in both soil and JB midgut suggests that the JB midgut archaeome may be environmentally sourced, with more tailored selection of the archaeome occurring in the JB hindgut. In turn, we found that JB larval infestation also increases the abundance of at least one methanogenic archaeon, *Methanobrevibacter*, in infested soil. Results underscore the potential impact of invasive root-feeding scarab larvae on the soil archaeome and highlight their potential contributions to climate change, especially in light of predicted global range expansion for this species.

## 1 Introduction

Global change can facilitate species invasions and, in turn, invasive species can amplify the deleterious impacts of global change (Sage, 2020). Since the Industrial Revolution, increasing atmospheric concentrations of methane (CH_4_) may be partly facilitated by anthropogenic ecological changes that favor the range expansion of CH_4_-producing arthropods (Hackstein and Stumm, 1994; Brune, 2019). Among insects, cockroaches, termites, and scarab beetle larvae are known to produce CH_4_, with the highest emission rates generally associated with termites (0–1,579 nmol CH_4_ × g body weight^−1^ × h^−1^) (Brune, 2019; Zhou et al., 2023) and scarab larvae (0–741 nmol CH_4_ × g body weight^−1^ × h^−1^) (Cazemier et al., 2003; Brune, 2019; Görres and Kammann, 2020; Avila-Arias et al., 2023). Numerous scarab beetles are important agricultural pests and the growing global distribution of some species may result from climate change (Kistner-Thomas, 2019). Beyond their negative impacts on plant growth and development (Johnson and Rasmann, 2015), the “feedback loop” between root-feeding scarab larvae and global climate change (Sage, 2020) lies in the capacity of larvae to alter soil structure and nutrient cycling through root herbivory, feces and cadaver inputs, and movement through the soil profile (Treonis et al., 2005; Frew et al., 2016; Gan and Wickings, 2020; MacLeod et al., 2024). In fact, activities commonly associated with soil-dwelling scarab larvae can increase soil microbial biomass, enhance the decomposition of organic matter, and increase soil greenhouse gas (GHG) emissions (Majeed et al., 2014; Kammann et al., 2017; Gan et al., 2018; Görres and Kammann, 2020; Avila-Arias et al., 2023). Although the ability of scarab larvae to increase GHG emissions from the soil has been documented, the mechanisms associated with CH_4_ production in scarab larvae are largely undetermined.

*Archaea* contribute to methane emissions via methanogenesis, the final step of organic matter decomposition in strictly anoxic habitats. *Archaea* are a phylogenetically and ecologically diverse group of microorganisms that inhabit various environmental and host-associated microbiomes (Borrel et al., 2020; Starke et al., 2021). In host-associated microbiomes, the abundance of methanogens in the gut often correlates positively to bacterial abundance, likely reflecting a symbiotic dependence on bacterial metabolism (Thomas et al., 2022). In CH_4_-emitting arthropods, methanogenesis has been linked to activities associated with the enlarged hindgut. It is fueled by hydrogen and reduced carbon compounds that are byproducts of the symbiotic digestion of organic matter, mainly in the form of lignocellulose and humus (Hackstein and van Alen, 2018; Brune, 2019). Archaeal diversity in the intestinal tract of arthropods known to emit methane has been identified via 16S rRNA-based surveys as members of the methanogenic orders *Methanobacteriales*, *Methanomicrobiales*, *Methanosarcinales*, and *Methanomassiliicoccales* (Hackstein and van Alen, 2018; Brune, 2019; Protasov et al., 2023). Despite these efforts, major prokaryotic groups have been severely undersampled, and the archaeome of arthropods remains poorly resolved (Protasov et al., 2023).

The Japanese beetle (*Popillia japonica* Newman; Coleoptera: Scarabaeidae) (JB) is an invasive insect that threatens both agricultural and urban landscapes and the biodiversity of invaded regions (Della Rocca and Milanesi, 2022a,b). Adult JB feeds on over 300 host species in 79 plant families (Potter and Held, 2002; Shanovich et al., 2019; Della Rocca and Milanesi, 2022a). The JB larval stage, which comprises ~80% of the JB life cycle, is concealed within the soil, thriving in areas where development is supported by large expanses of turf and pasture grasses (Potter and Held, 2002; Shanovich et al., 2019; Della Rocca and Milanesi, 2022a) and other agricultural landscapes. Due to its strong adaptability, this species has expanded rapidly in North America and more recently in mainland Europe (Poggi et al., 2022) with continuing expansion predicted in light of globalization, climate change and land-use conversion (Kistner-Thomas, 2019; Althoff and Rice, 2022; Della Rocca and Milanesi, 2022a). Further, the expansion of JB, and possibly other scarab species (Deans and Krischik, 2023), has the potential to amplify soil GHG emissions associated with climate change. Our previous study (Avila-Arias et al., 2023) demonstrated that JB larvae promote soil GHG release during infestation, both directly, through larval respiration and metabolism, and indirectly, through a syndrome of larval activities and behaviors that likely favor soil microbial activity (MacLeod et al., 2024). Laboratory experiments demonstrated that CH_4_ emissions were almost 7 times higher when larvae were imbedded in soil than when they were isolated from the soil. Furthermore, larval density was a significant predictor of CH_4_ emissions from soils under field conditions and from infested field soils under laboratory conditions, even after larvae were removed. While it is clear that soil GHG emissions are influenced by JB larval infestation, a linkage to changes to the soil microbiome has not been established.

Evidence for GHG-emitting archaea in the JB larval gut microbiome has not been well documented. Previous work describing JB gut microbiota using 16S rRNA gene amplicon sequencing (Avila-Arias et al., 2022) reported the presence of an amplicon sequencing variant (ASV) belonging to an order of archaeal methanogens, *Methanobrevibacter,* in the core microbiota of the JB larval hindgut. A non-methanogenic archaeon belonging to *Nitrososphaerales* in the core microbiota of the JB larval midgut and associated soils was also reported. However, other studies of the JB larval gut or associated soil did not report archaeal taxa or genes via omic surveys (Chouaia et al., 2019; Frias et al., 2023). Analysis of 16S rRNA gene sequencing data can generate inaccurate measure of archaeal diversity due to suboptimal selection of primers, PCR chimeras, and GC bias in rRNA operons (Shakya et al., 2013; Thijs et al., 2017; Abellan-Schneyder et al., 2021; Palkova et al., 2021; Thomas et al., 2022). These issues can overestimate the abundance of archaeal taxa and genes within the microbiomes of the insect digestive tract (Brauman et al., 2001; Brune, 2019), and soil (Starke et al., 2021). To overcome this, shotgun metagenome sequencing is an alternative option to analyze the taxonomic and functional profiles of the archaeome. This study explored the archaeome within the digestive system of third instar JB larvae and associated soil, using a whole-metagenome sequencing approach. Both taxonomic and functional aspects of the archaeome associated with the JB larval gut and host soil were considered. Because *Archaea* have been linked to methanogenesis, we hypothesized that archaeal communities and associated genes, specifically those related to methane emissions, may be abundant in the JB larval gut. We further discerned how JB larval infestation alters the soil archaeome. Based on the physicochemical changes in the soil due to JB larval activity (Gan et al., 2018; Avila-Arias et al., 2023; MacLeod et al., 2024), we predicted an archaeal footprint, where the relative abundance of archaeal taxa and functions associated with carbon and nitrogen cycling are more abundant in JB infested soils compared to uninfested soils.

## 2 Materials and methods

### 2.1 Field study

The third instar larva of the JB and their associated soil, as well as uninfested soil, were collected in October 2019, from a naturally infested location (Purdy Sod Farm, Lafayette, IN, United States) (Supplementary Figure S1). Soil at Purdy had a silty loam texture (22% sand, 58% silt, and 20% clay). Locations and other soil characteristics are presented elsewhere (Avila-Arias et al., 2022). Larvae and soil samples were recovered with aseptic techniques to minimize human/environmental DNA contamination.

To collect the larvae, turfgrass sod (Kentucky bluegrass, *Poa pratensis*, L.) displaying visual symptoms of infestation was pulled away from the soil and larvae were gently extracted from the soil by hand. Larvae were identified to species based on the conformation of the raster pattern using Richmond (2022) as a reference. After species confirmation, six individual larvae were transferred to individual wells within a 24-well, flat bottom, sterile plate (Corning® Costar®, Corning, NY, United States). Soil that was closely associated with each JB larva (i.e., soil lying within 1.0 cm of each larva) and uninfested soil (i.e., soil from an adjacent patch with visually healthy sod and no JB larvae) were also collected and transferred to individual wells of the sample plate. After sample collection, a sterile lid was placed back on the plate, and samples were transported to the laboratory in an insulated cooler. At the laboratory, soil was weighed, placed in DNA extraction buffer, and stored at −20°C until processed. Larvae were cleaned of soil particles and dissected as previously described (Avila-Arias et al., 2022). Briefly, soil particles were removed with a paintbrush, and larvae were flash-frozen at −20°C for 20 min. Larvae were then submerged in 70% ethanol for 10 min, before rinsing them again with 70% ethanol and sterile distilled water. To capture variability and ensure proper amount and quality of the DNA recovered, each JB gut sample consisted of gut contents from 2 contiguous JB larvae that were separately dissected, divided, placed in the same DNA extraction buffer and stored at −20°C until processed.

### 2.2 Manipulative laboratory experiment

A manipulative experiment was designed to test (i) how the soil archaeome is impacted by short-term JB infestation, and (ii) how the gut archaeome of third instar larvae collected from a naturally JB-infested location is altered by incubating those larvae in JB uninfested soil taken from a different location (i.e., experiment as main effect and experiment × compartment as interaction effect) (Supplementary Figure S1). For this purpose, a second set of larvae from the naturally infested location (Purdy) were collected, identified, and transported to the laboratory. At the laboratory, larvae were cleaned of host soil using a clean brush. Then, they were transferred to sieved (2 mm) soil collected from a JB-free location (Purdue University Nursery, West Lafayette, IN, United States). The Purdue nursery is approximately 6.54 km from Purdy, with soil at the nursery site having a sandier texture (sandy loam texture, 63% sand, 31% silt, and 6% clay). The nursery soil did not have a history of JB infestation, and no larvae were encountered during collection, so the soil was considered as “uninfested” for our purposes. Larvae collected at Purdy were placed into plastic bins containing nursery soil and maintained at room temperature for 48 h as a conditioning period (Avila-Arias et al., 2022). The conditioning period was imposed as a way to allow larvae to void their guts of previously consumed materials and become accustomed to the new soil. Conditioned larvae were transferred to microcosms containing fresh, sieved nursery soil (100 g dry weight) maintained at water holding capacity (WHC). Larvae were allowed to tunnel and feed within this soil for 96 h. The health of the larvae along with soil moisture, were checked daily. Unhealthy larvae were immediately replaced with new, healthy, conditioned larvae. After this period, soil samples and larvae were collected, prepared, and dissected as described above for the field study.

### 2.3 DNA extraction, metagenomic library generation, and HiSeq sequencing

Total genomic DNA was extracted from the prepared JB gut and soil samples using the DNeasy Power Soil Kit (Qiagen, Valencia, CA, United States) following the manufacturer’s instructions. DNA quality and purity were assessed by NanoDrop 2000 UV–Vis Spectrophotometer (Thermo Fisher Scientific Inc., Wilmington, DE, United States), using absorbance ratios of 260/280 nm (1.8–2.0) and of 260/230 nm (>1.7). DNA integrity was confirmed by electrophoresis in a 1% agarose gel with 1 × TAE buffer.

To capture variability and ensure proper amount and quality of the DNA recovered, genomic DNA from soils that were closely associated with the two JB larva used to prepare JB gut samples, were mixed to obtain single genomic DNA samples. Similarly, genomic DNA from uninfested soil consisted of a mix of two DNA extracted independently from contiguous locations. Genomic DNA extracted from the samples was stored at −20°C before amplification and sequencing.

A total of 4 sets of samples from each study (i.e., field study, manipulative laboratory experiment) were rendered, as follows: JB midgut, JB hindgut, JB infested soil, and uninfested soil, with three biological replicates of each (*n* = 24).

Library preparation and sequencing were performed by GENEWIZ LLC (South Plainfield, NJ, United States). The sequencing library was prepared using mechanical fragmentation and the NEBNext Ultra DNA (Illumina) library preparation method. Total community DNA was paired-end sequenced (2 × 100 bp) in a single lane of an Illumina HiSeq sequencing instrument (Genewiz, United States).

### 2.4 Bioinformatics and statistical analysis

Following their standard protocol, the shotgun raw sequences were annotated with Metagenomic Rapid Annotations using Subsystems Technology (MG-RAST) server v4.0.3 (Meyer et al., 2008). Briefly, assessment of the sample sequencing error based on artificial duplicate read measuring was achieved using duplicate read inferred sequencing error estimation (DRISEE) (Keegan et al., 2012). The MG-RAST pipeline uses a Bowtie2 aligner to remove sequences from unwanted genomes related to eukaryotic model organisms (Langmead and Salzberg, 2012). The sequences were annotated through blasting using BLAT (BLAST-like alignment tool algorithm) (Kent, 2002) against the M5NR databases, which provides nonredundant incorporation of different databases (Wilke et al., 2012). The taxonomic profile was constructed by Best Hit at E-value cutoff of 1 × 10^−15^, minimum alignment length of 50 base pairs, minimum percentage identity cutoff of 50 based on the NCBI’s reference sequence (RefSeq) database (Pruitt et al., 2007; Jiménez et al., 2015). Taxonomic features and their functional category for the archaeal community were retrieved using the RefSeq and KEGG Orthology (KO) database (Kanehisa et al., 2016), respectively. This data was used for the archaeal community’s diversity and relative abundance analyses. Archaeal diversity was analyzed using Qiime2 v 2020.2 (Bolyen et al., 2019) as detailed elsewhere (Avila-Arias et al., 2022). Analyses were carried out at the genus level for taxonomy and the function level for potential function. Sampling depth per sample was enough to provide robust comparisons, as revealed by rarefaction plots, with 2,178 and 1,271 reads for taxonomy and function, respectively, for JB-associated samples, and 37,233 and 20,781 reads for taxonomy and function, respectively, for soil associated samples.

Statistical analyses of α-diversity metrics were performed using R (version 3.6.1). The residuals’ normality and homogeneity of variance were tested using the Shapiro–Wilk test (stats-package) and Levene’s (car-package), respectively. To examine the influence of compartment (uninfested *vs*. infested soil OR midgut *vs*. hindgut), experiment (field *vs*. manipulative) and their interaction (compartment × experiment) on each α-diversity metric, either a two-way Analysis of Variance (ANOVA) using Tukey multiple comparisons of means as a *post hoc*, or the non-parametric approach of Aligned Rank Transform (ART) ANOVA (ARTool package) (Wobbrock et al., 2011; Kay and Wobbrock, 2019) were employed, depending on how well the residuals met the assumptions of the model.

DEICODE, a form of Aitchison Distance that is robust to high sparsity levels, was used to compare archaeal communities taxonomically and functionally across samples. For this analysis, factorial permutational analysis of variance (PERMANOVA, Adonis) with 999 permutations was performed using pooled data from field and manipulative experiments. Comparisons of archaeal communities between compartments were made using the model Y = experiment + compartment + (experiment × compartment). In contrast, comparisons between JB-infested and uninfested soils used the model Y = experiment + infestation + (experiment × infestation). When a significant interaction between the experiment and infestation or compartment was detected (α = 0.1), each experiment was subjected to separate analyses using compartment or infestation status as the independent variable at the same α-level (α = 0.1). Permutational analysis of multivariate dispersion (PERMDISP, 999 permutations) was also used to describe the homogeneity of dispersion among treatments. This analysis was performed first by pooling data from the two experiments using JB compartment or infestation status as independent factors. Separate analyses for each experiment were then performed using the same factors. Two additional methods were applied to investigate significant interactions between independent factors further. First, the EMPeror (Vázquez-Baeza et al., 2013) tool was also used to visualize 3D Principal Coordinate Analysis (PCoA) for DECOIDE. Next, between treatment differences in the relative abundance of archaeal taxa and functions per experiment were quantified through differential abundance analysis using DESeq2 (Weiss et al., 2017) in the MicrobiomeAnalyst (Dhariwal et al., 2017; Chong et al., 2020) platform.

Features that belonged to known methanogenic orders were selected to analyze methanogenic taxa. To analyze genes related to methanogenesis, all KOs associated with methanogenic pathways were selected and compared with the Kyoto Encyclopedia of Genes and Genomes (KEGG) reference pathway (Kanehisa et al., 2016). KOs were assigned to one of the four modules related to methanogenic pathways, which vary according to the substrate used: M00567 (H_2_ or CO_2_), M00357 (acetic acid decarboxylation), M00356 (methanol), and M00563 (methylamine, dimethylamine, and trimethylamine). The relative abundance of features was estimated by comparing the number of taxa or KOs assigned to a specific methanogenic taxa or module versus the number of features obtained per sample.

### 2.5 Data availability

Shotgun metagenome sequencing data from JB gut compartments and associated soil samples was deposited at the National Center for Biotechnology Information under the BioProject PRJNA868936. Quality-filtered and annotated metagenomes are available in MG-RAST with IDs mgm4872201.3 to mgm4872224.3.

## 3 Results

### 3.1 Metagenome sequencing, quality control and annotation

A total of 458,795,165 sequenced reads were recovered for the 24 samples (Supplementary Table S1), with the average number of sequenced reads (each *n* = 6) equaling 20,374,525 ± 2,066,607 for JB midgut, 18,845,650 ± 2,032,170 for JB hindgut, 16,684,254 ± 1,646,822 for uninfested soil and 20,561,431 ± 1,399,097 for JB infested soil. After passing quality control in MG-RAST, 86% of sequences with mean G + C content of 37 ± 10, 46 ± 11, and 64 ± 8, for midgut, hindgut, and soil, respectively, were used for downstream analyses (Supplementary Table S1). Three metagenomic samples (one hindgut sample from the manipulative laboratory experiment, one uninfested soil sample from the manipulative laboratory experiment and one uninfested soil sample from the field study) were excluded from further analysis because they rendered 2–3 orders of magnitude fewer taxonomic and functional annotations than the rest. Overall, 6.5 and 2.1% in the midgut, 34.6 and 11.3% in the hindgut, and 47.5 and 17.3% in the soil rendered hits for taxonomic and functional annotations, respectively (Supplementary Table S1). In general, a significant percentage of midgut sequences (69.6–76.3%) and most hindgut (98.9–99.1%) and soil (99.1–99.4%) sequences belonged to Prokaryotes. Of those, a relatively small number of sequences were annotated to *Archaea* in the midgut (0.3–0.6%), the hindgut (1.7–3.1%), and soil (0.5–0.8%).

### 3.2 JB gut associated archaeome

**Figure 1:**
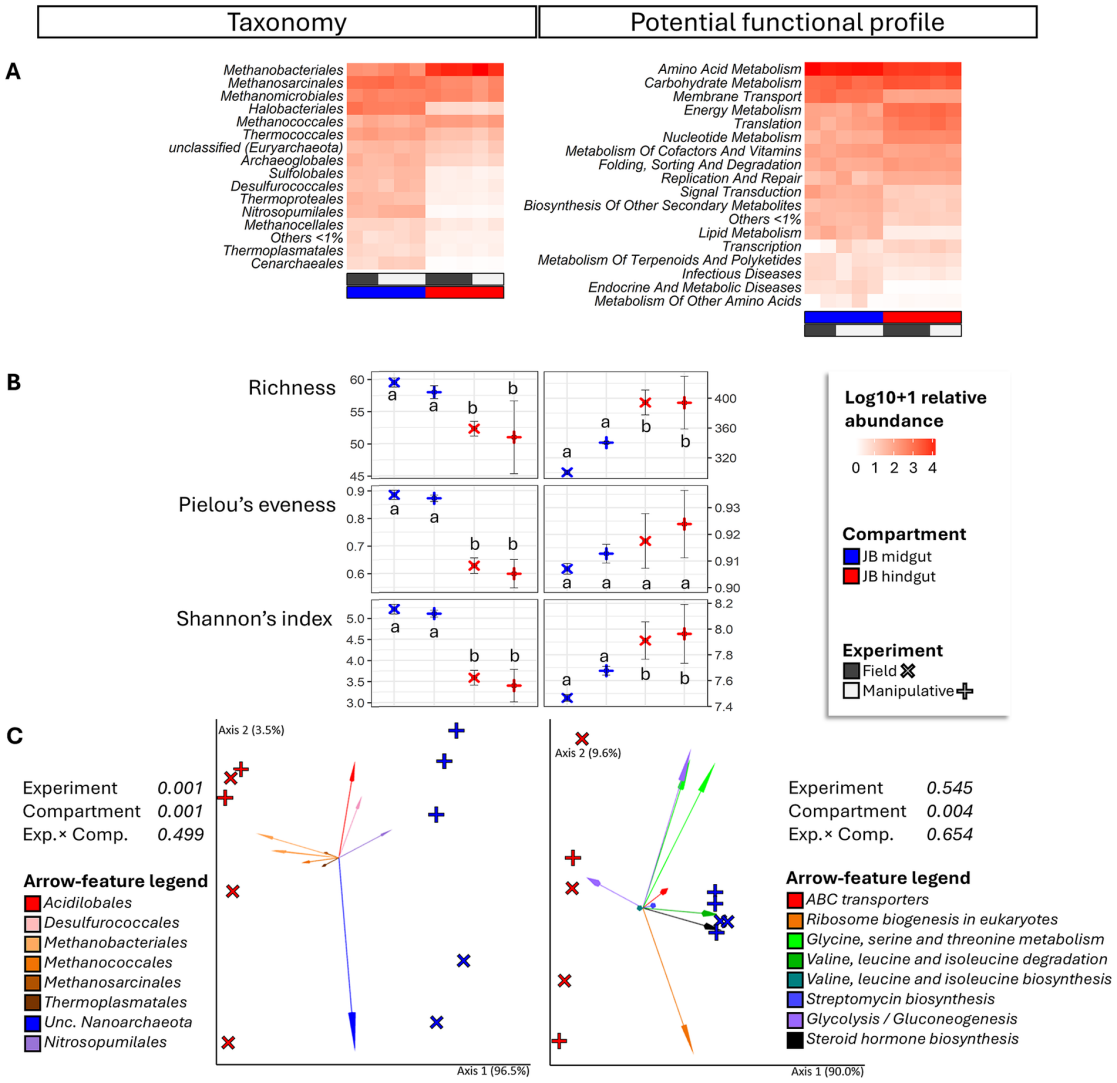
Heatmap **(A)**, alpha diversity **(B)** and compositional principal coordinate analysis (PCoA) biplots portraying beta diversity **(C)** of the archaeome in midgut and hindgut of third instar larvae of the Japanese beetle *Popillia japonica* Newman (JB). For the heatmap, microbiota composition is presented at the order level and potential functional composition is presented at level. The color ramp white to red indicates normalized (logarithm based 10 + 1) relative abundance of features. For alpha diversity, different letters represent significant differences (*p* < 0.05) by Tukey multiple comparisons of means or, in the case of taxonomical richness, by the Aligned Rank Transformed ANOVA. For beta diversity, the PCoA biplots were generated using DEICODE (Robust Aitchison PCA) (Martino et al., 2019) and visualized in Emperor (Vázquez-Baeza et al., 2013). Data points represent individual samples where symbol shape denotes experiment type while symbol color denotes gut compartment (i.e., midgut, hindgut). The top 10 features driving differences in ordination space in the PCoA are illustrated by the arrows. For taxonomy, the phyla are represented by specific hues, where *Crenarchaeota* (2) = red/pink, *Euryarchaeota* (6) = orange/brown, *Nanoarchaeota* (1) = green, and *Thaumarchaeota* (1) = blue. For potential function, function at level 1; level 2 are represented by specific hues, where Environmental information processing; Membrane transport (1) = red, Genetic information Processing; Translation (1) = orange, Metabolism; Amino acid metabolism (4) = green, Metabolism; Biosynthesis of other secondary metabolites (1) = blue, Metabolism; Carbohydrate metabolism (2) = purple, and Metabolism; Lipid metabolism (1) = black. Analyses were carried out at the genus level for taxonomy and the function level for potential function. Sequences were obtained using shotgun metagenome sequencing (HiSeq Illumina), analyzed using MG-RAST (Keegan et al., 2016), and annotated using the RefSeq (O’Leary et al., 2015) or KO (Kanehisa et al., 2016) databases. For taxonomic affiliation at order level refer to Supplementary Table S2. For function annotation at level 1 refer to Supplementary Table S3.

Although there was some variability in the archaeal community among the midgut samples, overall patterns were discernable (Figure 1A). *Euryarchaeota* was the most abundant archaeal phylum (84.3%), with *Halobacteriales*, *Methanosarcinales*, *Methanobacteriales*, and *Methanomicrobiales* representing abundances >12%. Phyla *Crenarchaeota* and *Thaumarchaeota* were also present in the midgut, with *Sulfolobales* and *Nitrosopumilales* representing the most abundant orders, respectively. Archaeal functional composition in the midgut mainly consisted of amino acid metabolism, membrane transport, and carbohydrate metabolism. Other abundant (>3%) functions in the midgut were related to genetic information processing (translation, and folding, sorting and degradation), metabolism (energy, cofactors and vitamins, nucleotide), and environmental information processing (signal transduction).

In the hindgut, the phylum *Euryarchaeota* was also the most abundant Archaeal constituent (97.8%), with *Methanobacteriales* (58%), *Methanomicrobiales* (12.4%), and *Methanosarcinales* (11.6%) representing the most abundant orders (Figure 1A). Archaeal functional composition in the hindgut was mainly (>10%) represented by metabolism (amino acid, carbohydrate, and energy), and translation. Other abundant (>3%) archaeal functions associated with the hindgut included metabolism (nucleotide, cofactors and vitamins), genetic information processing (folding, sorting and degradation, and replication and repair), and environmental information processing (replication and repair).

Alpha diversity of archaeal microbiota associated with the midgut was higher than that of the hindgut under both experimental conditions (i.e., field and manipulative) (Figure 1B). Both gut compartment (*F* = 16.85, *p* = 0.001, *R*^2^ = 49.2%) and experimental condition (*F* = 10.5, *p* = 0.001, *R*^2^ = 30.7%) were significant predictors of beta diversity of archaeal taxa (Figure 1C). Regarding potential function, the archaeal community in the hindgut exhibited greater richness and diversity (Shannon index) compared to the midgut (Figure 1B). The gut compartment significantly influenced the beta diversity of archaeal potential functions (*F* = 6.7, *p* = 0.004, *R*^2^ = 47.9%) but the experimental conditions did not (Figure 1C).

**Figure 2:**
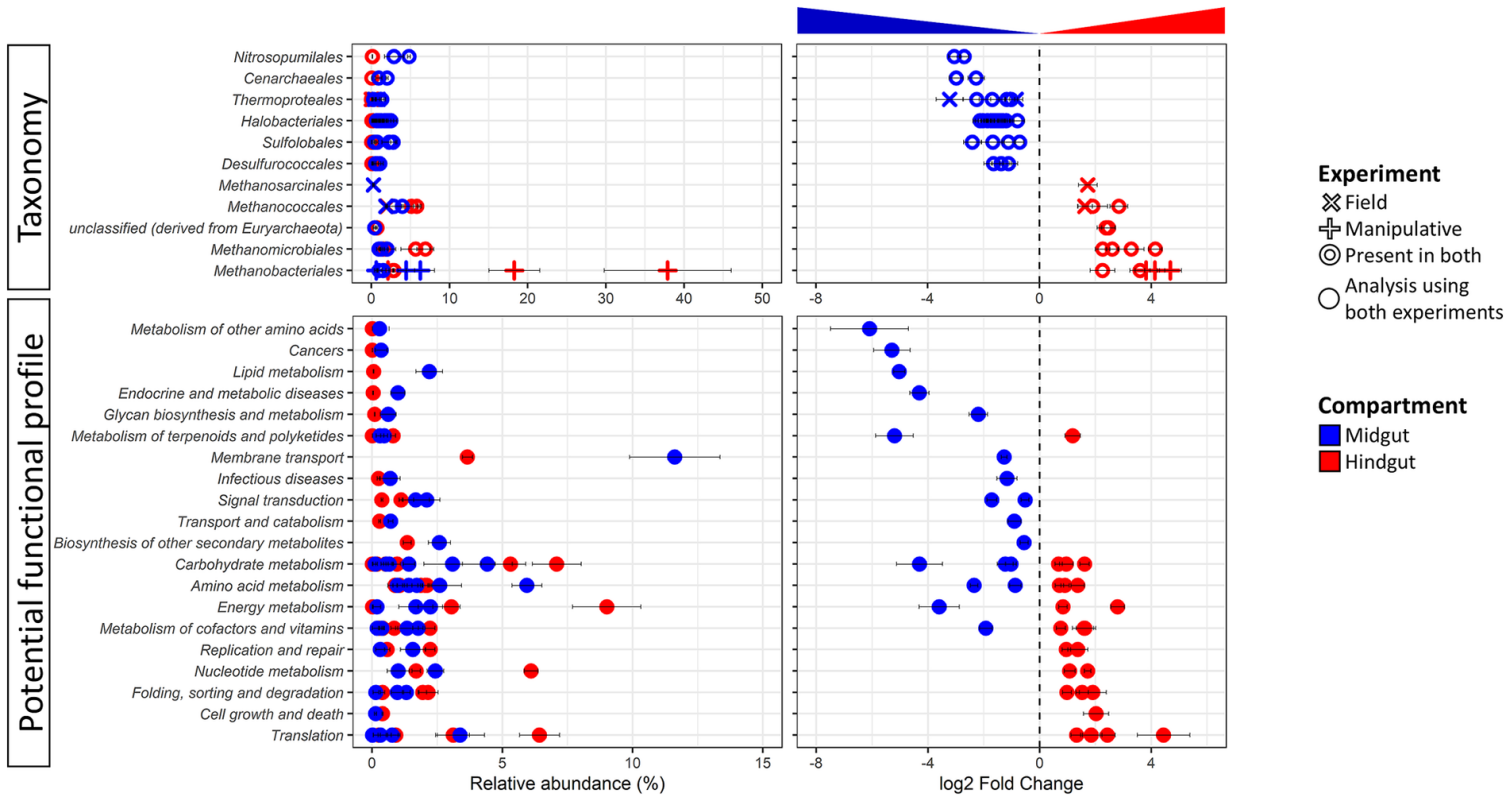
Differentially abundant taxa (top panel) and potential function (bottom panel) of the Japanese beetle *Popillia japonica* Newman (JB) midgut (blue) *vs.* hindgut (red). Relative abundance (left panel) and logarithmic scale base 2 (log2) fold change (right panel) for differentially abundant (adjusted *p*-value FDR < 0.005, 95% confidence intervals) features based on DESeq2 (Love et al., 2014) are presented. A negative log2 Fold Change indicates higher abundance of a given feature in the midgut while a positive log2 Fold Change indicates higher abundance in the hindgut. Thus, further from 0 the log2 Fold Change value, the greater the difference in the relative abundance of a given feature between midgut and hindgut. The symbol for taxa represents features at the genus level that were differentially abundant in either or both experimental conditions. The symbol for potential function represents features at level 3 that were differentially abundant independent of the experimental condition since only compartment was significant in beta diversity analysis (DEICODE, *p*-value for gut compartment = 0.004). For detailed information regarding taxa at the genus level and potential function at the KO level refer to Supplementary Table S4.

Passage through the alimentary canal from midgut to hindgut resulted in changes in the relative abundance of at least 27 archaeal taxa within the Orders *Euryarchaeota*, *Crenarchaeota*, and *Thaumarchaeota* (Figure 2; Supplementary Table S4A). Among the archaeal taxa with greater relative abundance in the JB midgut regardless of experimental conditions, those belonging to the phyla *Euryarchaeota* (11 taxa within the family *Halobacteriaceae*), *Crenarchaeota* (6 taxa including *Metallosphaera* sp., *Vulcanisaeta* sp., and *Aeropyrum* sp.), and *Thaumarchaeota* (2 taxa including *Cenarchaeum* sp. and *Nitrosopumilus* sp.) comprised the majority. Of the archaeal taxa that were more abundant in the JB hindgut, most (≥63%) displayed a similar pattern regardless of experimental conditions. All taxa with greater relative abundance in the JB hindgut belonged to the phylum *Euryarchaeota*, with the greatest increases represented by *Methanocorpusculum* sp. (*Methanomicrobiales*) in both experiments.

Concomitant potential functional differences in the JB gut microbiome were also observed with the relative abundance of 50 functions differing between gut compartments (Figure 2; Supplementary Table S4B). Differentially abundant functions for *Archaea* were distributed across several functional categories, suggesting that differences in potential metabolic capabilities between the gut compartments comprise a broad range of intracellular and extracellular processes. Of those, 24 functions were significantly higher in the JB midgut, with most (16) being involved in metabolism with the greatest differences related to glutathione metabolism (Metabolism of other amino acids), drug metabolism (Xenobiotics biodegradation and metabolism), geraniol degradation (Metabolism of terpenoids and polyketides), and steroid hormone biosynthesis (Lipid metabolism). The relative abundance of 26 archaeal functions were significatively higher in the JB hindgut compared to the midgut. Of these, the relative abundance of most (15) archaeal functions that were more abundant in the JB hindgut were related to metabolism, with the greatest differences related to methane and nitrogen metabolism (both within Energy metabolism). The relative abundance of 10 functions related to genetic information processing were also significantly higher in the hindgut, with mRNA surveillance pathway (Translation) showing the greatest difference.

**Figure 3:**
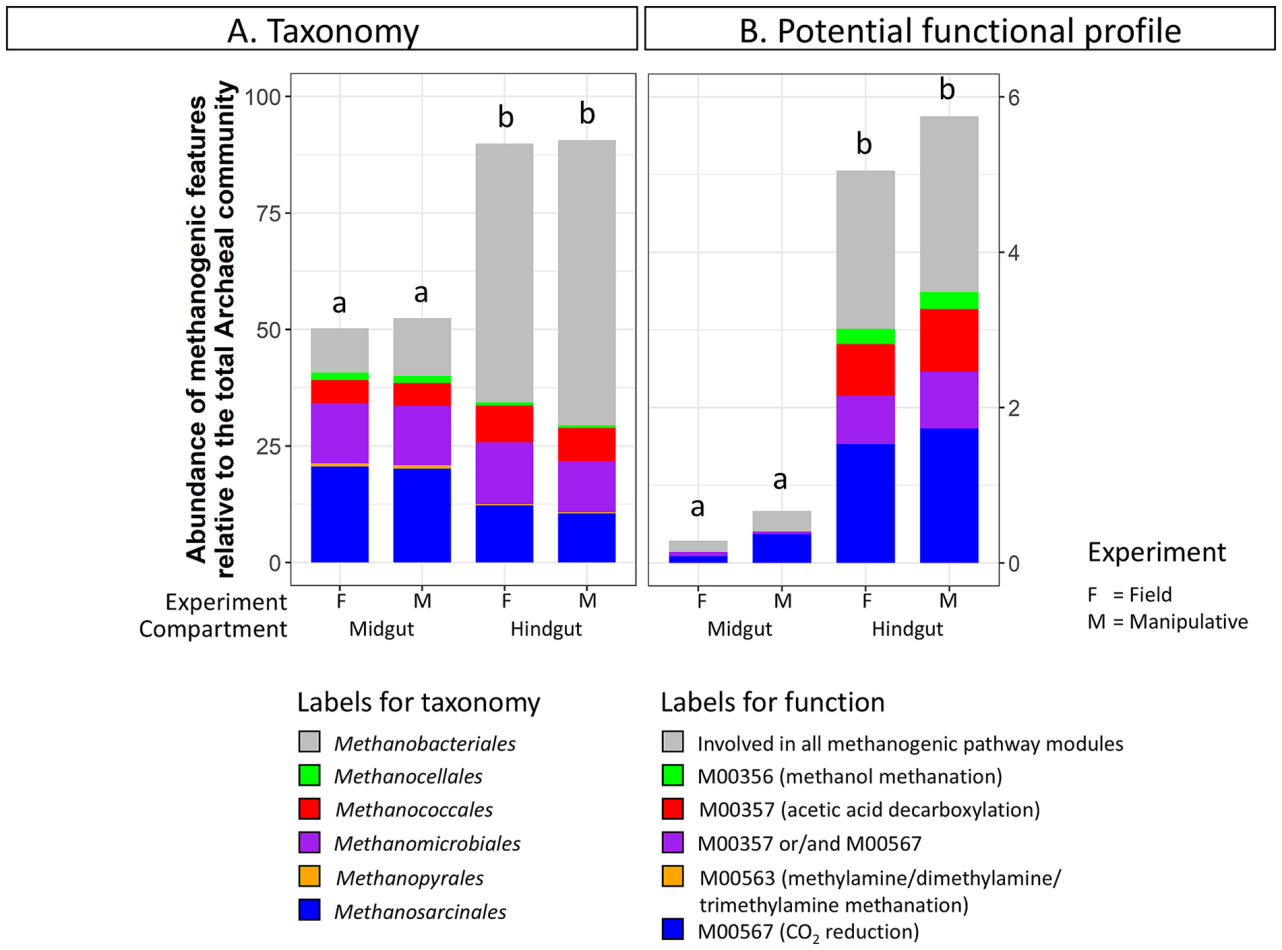
Abundance of **(A)** methanogenic taxa (order level) and **(B)** methanogenic potential function (pathway modules) relative to the total Archaeal community in the gut compartments (i.e., midgut, hindgut) of third instar larvae of the Japanese beetle *Popillia japonica* Newman (JB). For detailed information regarding taxa at the genus level and potential function at the function level refer to Supplementary Table S5.

Taxa and potential functions associated with methane metabolism showed some of the most significant changes between the JB midgut and hindgut compartments. The abundance of methanogenic taxa significantly increased from the midgut (52 ± 3%) to the hindgut (90 ± 2%) in both experiments (Figure 3A). Several genera within the Orders *Methanobacteriales*, *Methanococcales*, *Methanomicrobiales*, and *Methanosarcinales* appeared to be significantly more abundant in the hindgut compared to the midgut (Supplementary Table S5A). Similarly, the relative abundance of methane metabolism related genes increased from the midgut (1.7 ± 0.7%) to the hindgut (9.0 ± 1.3%). Of those, the abundance of potential functions related to methanogenic pathway modules increased from the midgut (0.6 ± 0.3%) to the hindgut (5.3 ± 0.7%) (Figure 3B), with potential functions within the methanogenic pathway modules M00567 (CO_2_ reduction), M00357 (acetic acid decarboxylation), and M00356 (methanol methanation) increasing significantly (Supplementary Table S5B).

### 3.3 Soil archaeome and changes due to JB larval infestation

**Figure 4:**
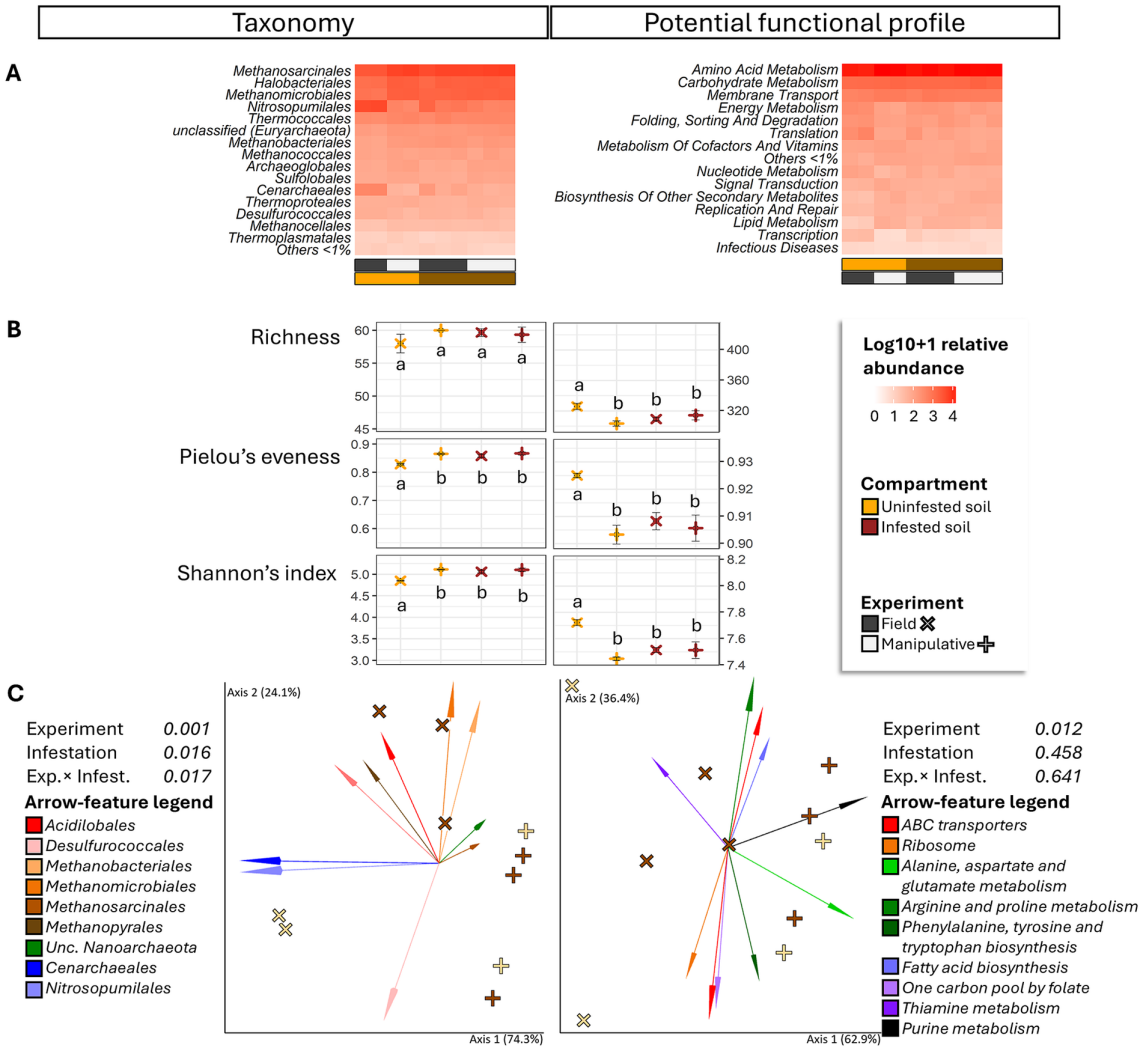
Heatmap **(A)**, alpha diversity **(B)** and compositional principal coordinate analysis (PCoA) biplots portraying beta diversity **(C)** of the archaeome in soils collected from uninfested areas and areas impacted by third instar larvae of the Japanese beetle *Popillia japonica* Newman (JB). For the heatmap, microbiota composition is presented at the order level and potential functional composition is presented at level. The color ramp white to red indicates normalized (logarithm based 10 + 1) relative abundance of features. For alpha diversity, different letters represent significant differences (*p* < 0.05) by Tukey multiple comparisons of means or, in the case of taxonomical richness, by the Aligned Rank Transformed ANOVA. For beta diversity, the PCoA biplots were generated using DEICODE (Robust Aitchison PCA) (Martino et al., 2019) and visualized in Emperor (Vázquez-Baeza et al., 2013). Data points represent individual samples where symbol shape denotes experiment type while symbol color denotes infestation level (i.e., uninfested, infested). The top 10 features driving differences in ordination space in the PCoA are illustrated by the arrows. For taxonomy, the phyla are represented by specific hues, where *Crenarchaeota* (2) = red/pink, *Euryarchaeota* (6) = orange/brown, *Nanoarchaeota* (1) = green, and *Thaumarchaeota* (1) = blue. For potential function, function at level 1; level 2 are represented by specific hues, where Environmental information processing; Membrane transport (1) = red, Genetic information Processing; Translation (1) = orange, Metabolism; Amino acid metabolism (4) = green, Metabolism; Biosynthesis of other secondary metabolites (1) = blue, Metabolism; Carbohydrate metabolism (2) = purple, and Metabolism; Lipid metabolism (1) = black. Analyses were carried out at the genus level for taxonomy and the function level for potential function. Sequences were obtained using shotgun metagenome sequencing (HiSeq Illumina), analyzed using MG-RAST (Keegan et al., 2016), and annotated using the RefSeq (O’Leary et al., 2015) or KO (Kanehisa et al., 2016) databases. For taxonomic affiliation at order level refer to Supplementary Table S2. For function annotation at level 1 refer to Supplementary Table S3.

To understand how JB larval invasion influences the soil archaeome, we compared the microbiome of uninfested soil to JB-infested soil by pooling samples collected from both field and laboratory experiments. Independent of the infestation status, soils from the field (Figure 4A) were comprised primarily (>10%) by archaeal taxa belonging to *Thaumarchaeota* (order *Nitrosopumilales*) and *Euryarchaeota* (orders *Methanosarcinales*, *Halobacteriales*, and *Methanomicrobiales*). Archaeal composition in soils from the manipulative laboratory experiment followed similar patterns regardless of the infestation status, with the most abundant archaeal taxa (>10%) belonging to *Euryarchaeota* (orders *Methanosarcinales*, *Halobacteriales*, and *Methanomicrobiales*).

Independent of infestation status or experimental conditions, the most abundant functions (>3%) in soils appeared to be mainly represented by metabolisms (amino acid, >31%; carbohydrate, >13%; energy, >4%; nucleotide, >3%; and cofactors and vitamins, >4%), environmental information processing (membrane transport, >8%; and signal transduction, >3%), and genetic information processing (translation, >4%; and folding, sorting and degradation, >4%) (Figure 4A).

JB larval infestation resulted in significant but differential effects on taxonomic and functional alpha-diversity of the soil archaeome depending on the circumstances of the infestation (field *vs*. manipulative) (Figure 4B), with lower taxonomic, but higher functional alpha diversity in uninfested soil compared to infested soil in the field. Similarly, the influence of JB larval infestation on the β-diversity of archaeal taxa (Figure 4C) varied with experimental conditions (experiment × infestation interaction, *F* ≥ 6.38; *p* = 0.017, *R*^2^ = 20.7%), with JB infestation having a more pronounced effect under field conditions. Only the experimental condition (*F* = 4.72, *p* = 0.012, *R*^2^ = 38.3%) was a significant predictor of functional beta diversity.

**Figure 5:**
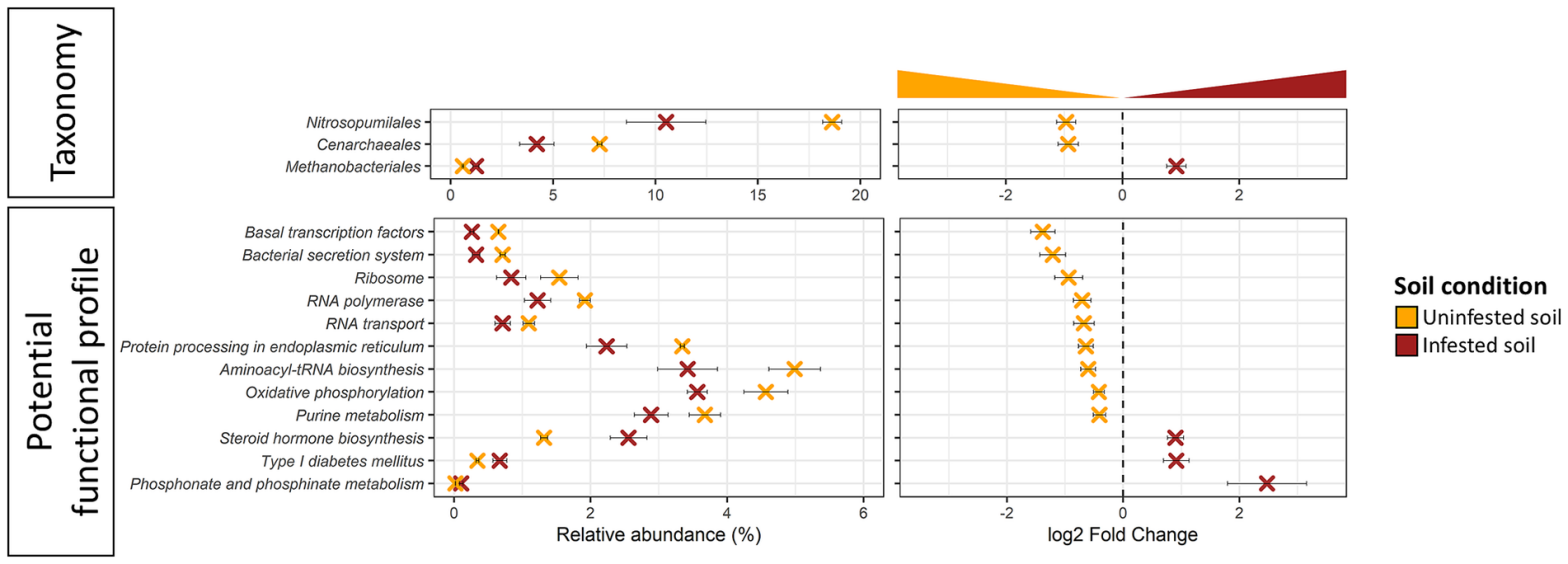
Differentially abundant taxa (top panel) and potential function (bottom panel) of uninfested soil (orange) *vs.* soil infested with the Japanese beetle *Popillia japonica* Newman (JB) (brown) in samples from the field experiment. Soils from the laboratory experiment showed no differentially abundant features. Relative abundance (left panel) and logarithmic scale base 2 (log2) fold change (right panel) for differentially abundant (adjusted *p*-value FDR < 0.005, 95% confidence intervals) features based on DESeq2 (Love et al., 2014) are presented. A negative Log2 Fold Change indicates higher abundance of a given feature in uninfested soil while a positive Log2 Fold Change indicates higher abundance in the JB infested soil. Thus, further from 0 the log2 Fold Change value, the greater the difference in the relative abundance of a given feature between uninfested soil and JB infested soil. Microbiota composition is presented at the Order level and potential functional composition is presented at level 3. For detailed information regarding taxa at the genus level and potential function at the KO level refer to Supplementary Table S6.

JB infestation altered (DeSeq2, Log2FC ≠ 0, FDR ≤ 0.005) the representation of 3 genera of *Archaea* in the field (Figure 5; Supplementary Table S6A), while no significant changes were observed in the short-term under manipulative conditions. Of the altered taxa, two genera (*Thaumarchaeota*: *Cenarchaeum* sp. and *Nitrosopumilus* sp.) were lower while one genus (*Euryarchaeota*: *Methanobrevibacter* sp.) was higher in soils infested with JB larvae. Similarly, taxonomic changes in the soil resulting from JB infestation also resulted in the differential abundance of 12 potential archaeal functions (Figure 5; Supplementary Table S6B). Differentially abundant archaeal functions resulting from JB infestation included increases in those related to the metabolism of other amino acids (phosphonate and phosphinate), and lipids (steroid hormone). In contrast, the relative abundance of 9 Archaeal functions decreased as a result of JB infestations, including several related to genetic information processing, metabolism, and environmental information processing.

**Figure 6:**
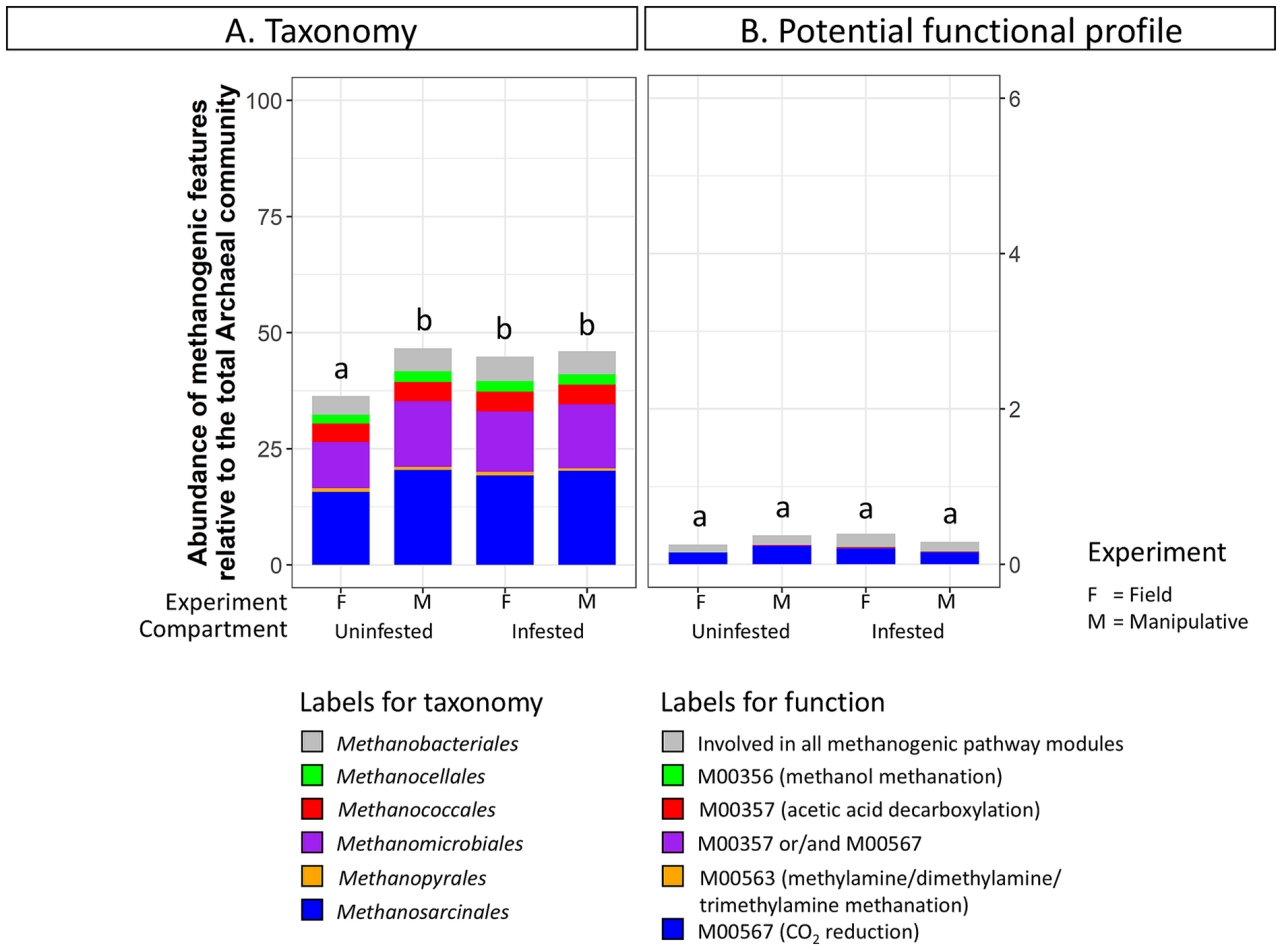
Abundance of **(A)** methanogenic taxa (order level) and **(B)** methanogenic potential function (pathway modules) relative to the total Archaeal community in uninfested soil and soil infested with the Japanese beetle *Popillia japonica* Newman (JB). For detailed information regarding taxa at the genus level and potential function at the function level refer to Supplementary Table S7.

Methanogenic archaeal taxa were more abundant in infested soil (44.86 ± 1.2%) compared to uninfested soil (36.3 ± 0.4%) under field conditions (Figure 6; Supplementary Table S7). In contrast, no discernable changes in the relative abundance of methanogenic taxa due to JB larval infestation were observed in soils from the shorter-term manipulative experiment*. Methanobrevibacter* was significantly more abundant in infested soils (DESeq2, *p* < 0.05) under field conditions. No significant changes in methanogenic related function were detected in soil under either experimental condition due to JB infestation.

## 4 Discussion

This study examined the taxonomic and functional profiles associated with microbiological methane production in larvae of the invasive Japanese beetle *Popillia japonica* Newman (JB) and the soils they infest. Previous efforts (Avila-Arias et al., 2023) revealed that JB larvae promote methane emissions from the soil both directly through larval metabolic activities (i.e., respiration, intrinsic metabolism) and indirectly by creating conditions in the soil that favor GHG-associated microbial activity (Avila-Arias et al., 2023). In the present study, we used shotgun metagenome sequencing and a gene-centric approach, that is database dependent, to confirm the presence of *Methanobacteriales* (*Euryarchaeota*) previously identified using a 16S rRNA gene sequencing survey (Avila-Arias et al., 2022), along with several other methanogens in the JB larval digestive tract. Most of the archaeal taxa detected in the JB gut archaeome were methane producers belonging to the *Euryarchaeota*. In parallel with the above investigations, potential functions within three methanogenic pathway modules were identified within the JB digestive tract.

### 4.1 Methanogenesis: an important archaeal function in the hindgut

**Figure 7:**
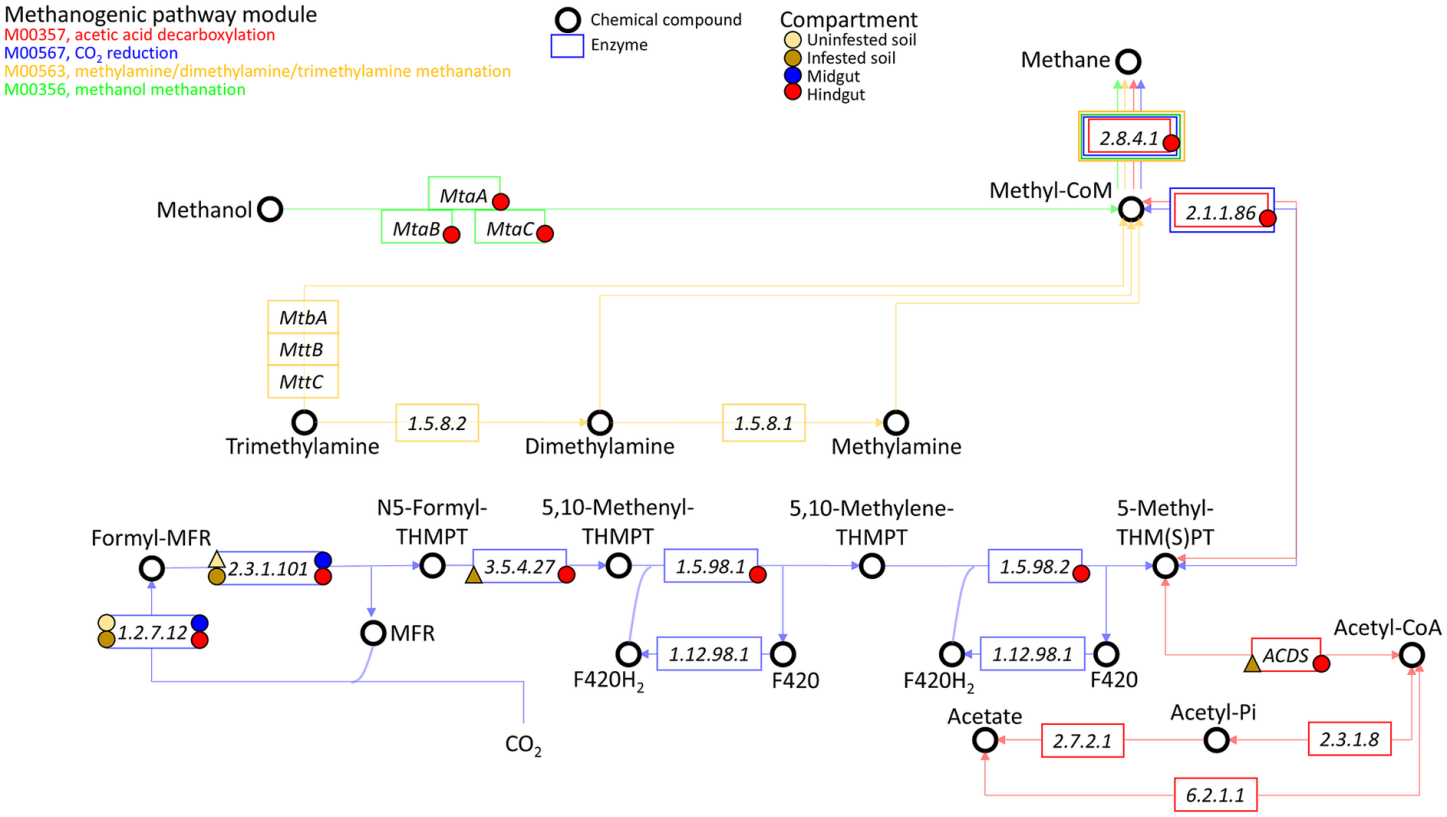
Presence of genes related to methanogenesis in the archaeome of the gut compartments (i.e., midgut, hindgut) of third instar larvae of the Japanese beetle *Popillia japonica* Newman (JB), uninfested soil, and soil infested with JB. The four known methanogenic pathway modules are shown: M00357 (acetic acid decarboxylation), M00567 (CO_2_ reduction), M00563 (methylamine/dimethylamine/trimethylamine methanation), and M00356 (methanol methanation). Presence of genes associated with the enzyme represented within the rectangles are indicated by the symbol, where a circle represents presence in ≥2 replicates while a triangle represents presence in only 1 of the replicates. The color of the symbol represents the compartment in which the gene was observed.

The enriched methanogenic taxa and methane metabolism related sequences in the JB hindgut provide strong evidence that methanogenesis is an important archaeal function and aligns with previous reports of other scarab beetle larvae, termites, and cockroaches (Brune, 2019). Methanogenic archaea are strict anaerobes, thriving where terminal electron acceptors such as oxygen, nitrate, iron(III), and sulfate are absent or rapidly depleted (Brune, 2019). Thus, the growth and proliferation of methanogens is largely limited to anaerobic environments, such as water, sediments, soils, and the digestive tracts of ruminants, humans, and insects (Gaci et al., 2014; Brune, 2019; Gurung et al., 2019; Kim et al., 2020; Klammsteiner et al., 2020; Scharf and Peterson, 2021). Methanogens have a terminal position in microbial trophic interactions as they use a limited number of one-carbon compounds (e.g., carbon dioxide, carbon monoxide, methanol, methylamines, and methyl sulfides) and acetate as electron acceptors resulting from anaerobic degradation of organic matter by hydrolytic and fermentative bacteria. In scarab beetle larvae, methanogens are apparently attached to the gut epithelium or to tree-like epithelial invaginations formed within the fermentation sac in the hindgut (Brune, 2019). Based on some potential functions revealed by our metagenomic analysis, methanogenesis appears to be performed in the hindgut of third instar JB larvae, mainly via CO_2_ reduction and methanol methanation (Figure 7; Supplementary Table S5C).

While genes involved in all methanogenic pathway modules accounted for ~40% of all related methanogenesis genes in the JB hindgut, genes exclusively related to CO_2_ reduction (or hydrogenotrophic methanogenesis) represented at least ~30% of possible methanogenesis pathways in the hindgut. Hydrogenotrophic methanogens use H_2_ or formate as electron donors, reductants that are produced in the midgut during the fermentative breakdown of organic matter, and transported to the hindgut via the hemolymph (Lemke et al., 2003; Brune, 2019). The high relative abundance of taxa that includes hydrogenotrophic methanogens, such as the orders *Methanobacteriales*, *Methanococcales*, *Methanomicrobiales*, and *Methanosarcinales*, in the hindgut, support the hypothesis of methane being produced via the CO_2_ reduction pathway in this compartment.

Methylotrophic methanogenesis is methane formation from methylated compounds such as methanol, methylamines or methylated thiols. This study identified the three genes involved in the methanol methanation pathway in the JB hindgut. While there was no evidence of functions related to the use of methylamine compounds within the JB gut, the methanol methanation pathway represented at least ~4% of all potential methanogenic pathways. *In vitro* assays using the larval hindgut of the scarab beetle *Pachnoda ephippiata* (Lemke et al., 2003) showed that the stimulation of methanogenesis by methanol was much higher than that caused by H_2_. However, under *in vivo* anaerobic conditions, methanol availability for methanogenesis could be limited because methanol also serves as a carbon source and electron donor for other metabolic processes such as denitrification, acetogenesis, and sulfate reduction (Fischer et al., 2021; Bueno de Mesquita et al., 2023). This partially explains the relatively low abundance of genes related to the methanol methanation pathway compared to hydrogenotrophic methanogenesis within the JB hindgut. Taxonomically, methylotrophic methanogenesis is generally performed by members of the family *Methanosarcinaceae* (within *Methanosarcinales*) (Oren, 2014; Lyu and Liu, 2019). Although the relative abundance of *Methanosarcinaceae* in the hindgut (9.9 ± 1.1%) appeared to be lower compared to that in the midgut (17.8 ± 0.2%), one genus in particular, *Methanohalobium,* was more abundant in the hindgut. Commonly found in hypersaline environments, this methylotrophic methanogen is extremely halophilic and can accumulate intracellular potassium at high concentrations in the cytoplasm (Oren, 2014; McGenity and Sorokin, 2018; Protasov et al., 2024). To our knowledge, this is the first report highlighting its presence in the digestive tract of an organism.

In examining the acetic acid decarboxylation pathway, the only detected function was the acetyl-CoA decarbonylase/synthase (ACDS) multienzyme complex, which was found exclusively in the hindgut. This complex accounted for approximately 13% of the potential methanogenic pathways in the JB hindgut. In methanogens, the primary role of the ACDS complex is to cleave acetate into methane and CO₂ for energy production. However, it can also operate in reverse, synthesizing acetyl units for carbon assimilation during autotrophic growth on C1 substrates (Grahame et al., 2005). The presence of this complex has been reported in *Methanosarcina* and *Methanosaeta*, both within the order *Methanosarcinales*, as well as in some non-methanogenic archaea (Dai et al., 1998). Given that this was the only enzyme associated with acetic acid decarboxylation for methanogenesis that we detected, and considering the lower abundance of *Methanosarcinales* in the hindgut compared to the midgut, these findings suggest that acetic acid decarboxylation is likely not a major methanogenic pathway in the JB hindgut. This conclusion aligns with previous studies that found little evidence for aceticlastic methanogenesis in insect guts (Brune, 2019).

### 4.2 Natural JB larval infestations alter soil archaeal diversity

JB infestation increased the relative abundance of a methanogenic archaeon, *Methanobrevibacter* (*Methanobacteria*, *Euryarchaeota*) in field soils. However, our manipulative experiment, which included the artificial infestation of homogenized soil with JB larvae for ~100 h, did not mirror this result. This finding is on par with previous work (Avila-Arias et al., 2023), indicating a significant, JB-density-dependent CH_4_ footprint from field soils with a history of natural JB infestation, but not from short-term artificial infestation of soils in the lab. Infested soils collected from the field hosted JB larvae for ~3 months prior to the time when our samples were collected. These soils also had a history of natural, high-density JB infestations for at least two consecutive years. Naturally occurring, longer-term and recurrent infestation by JB larvae thus appears to carry with it an increase in (i) the magnitude of disturbance, resulting in significant increases in CH_4_ emissions (Avila-Arias et al., 2023) and (ii) the relative abundance of at least one methanogenic archaeon in infested soils.

Although the relative abundance of *Methanobrevibacter* in JB infested field soils reached ~1.2%, no methanogenic related potential function was more abundant in infested soils than in uninfested soils. Previous studies have highlighted the limitations of shotgun metagenome sequencing in investigating specific archaeal metabolic pathways or potential functions in highly microbial diverse environments such as the soil food web (D'Alò et al., 2023). Metagenomic sequencing reveals microbial taxa and functional gene information, including DNA from microbes with widely varying physiological states (Jansson and Hofmockel, 2018). In fact, using a proteomics approach on soil across global biomes, Starke et al. (2021) reported a relatively large proportion (2.3%) of a protein that is central to methanogenic pathways, the methyl-coenzyme M reductase, which was not elucidated via metagenomics (D'Alò et al., 2023). As such, future efforts to elucidate differences in soil community function and activity due to JB larval infestation may require other multi-omics approaches, such as metatranscriptomics, to identify active functions, metaproteomics to determine functional capacity and identify the proteins present, and metabolomics to quantify the end products of microbial activity.

### 4.3 JB midgut archaeome appears to be environmentally sourced for digestion and nutrient uptake

Similar patterns of high abundance for methanogens were observed in the JB midgut and host soil. The only methanogenic order that was consistently more abundant in the midgut compared to those in the soil was *Methanobacteriales*, but taxa within *Methanobacteriales* were most abundant in the hindgut. Members of the *Methanobacteriales* are the most common archaeal lineage in the intestinal tract of terrestrial arthropods (Borrel et al., 2020; Protasov et al., 2023), which likely acquire these microbes through contact with and ingestion of soil.

Aside from methanogens, the midgut of JB larvae hosts diverse archaeal taxa, including *Halobacteriales* (*Euryarchaeota*), *Desulfurococcales*, *Sulfolobales and Thermoproteales* (*Crenarchaeota*), and *Nitrosopumilales* and *Cenarchaeales* (*Thaumarchaeota*). These archaeal clades are likely environmentally acquired, since they were also found in the soil. They have been associated with genes for lignocellulose and hydrocarbon degradation, processes crucial for breaking down complex plant materials—a key adaptation for root herbivory (Cragg et al., 2015; Prasad et al., 2018; Somee et al., 2022). *Halobacteriales*, typically halophilic microorganisms, have adapted to the JB midgut environment likely due to the high pH (Swingle, 1931; Chouaia et al., 2019) and possibly large concentrations of potassium ions in this gut compartment as seen in other insects (Purdy, 2007; Wada et al., 2020). *Crenarchaeota* contribute through sulfur cycling and aromatic compound degradation (Baker et al., 2020; Somee et al., 2022), while *Thaumarchaeota*, possibly positioned near oxygenated areas of the gut, may play roles in nitrogenous waste removal and vitamin synthesis (Martino et al., 2019; Haber et al., 2021). Potential functional analysis of the midgut microbiome reveals enrichment in membrane transport, amino acid metabolism, and carbohydrate metabolism, with high activity of ATP-binding cassette (ABC) transporters that facilitate nutrient uptake under alkaline conditions. These functions not only enhance digestion, detoxification, and oxidative stress responses, but also enable JB to efficiently exploit plant roots as a food source, bolstering their adaptability and invasiveness in new environments. Such traits underline the importance of the gut microbiome in supporting JB’s success as a destructive root herbivore and a globally invasive species.

Previous efforts using multiple locations across Indiana and Wisconsin (United States), support the correlation between soil microbial communities and the JB gut micro- and mycobiota (Avila-Arias et al., 2022; Avila-Arias et al., 2023). Particularly in the midgut, core microbial communities were less defined and more similar to the host soil, consistent with a region in transition between the soil and hindgut. However, core microbiota in the hindgut were more tightly defined, reflecting a smaller subset of the soil microbial community. Similar to other soil-dwelling scarabs, it is likely that the unique conditions of the JB larval gut (Chouaia et al., 2019) provides microenvironments suitable for microbial recruitment from the host soil (Lemke et al., 2003; Huang et al., 2010), including those of the archaeal kingdom.

### 4.4 Future directions

Understanding the gut microbiome of insects facilitates the study of host adaptation to complex environments and, in the present study, provides mechanistic insights into a potential climate change feedback loop associated with methane production in a globally expanding invasive species. Soil-dwelling scarab larvae are naturally in close contact with the rich and diverse reservoir of microbes in the soil. As such, these scarabs harbor an ecologically rich and taxonomically diverse assemblage of gut microbes. However, the contributions of most of these microbes to digestive physiology and nutritional ecology continue to be relatively uncharacterized. To achieve this, an understanding of gut microbiome acquisition (maternal *vs*. environmental), identification of potential novel microbial species and genes, the differentiation of viable and active taxa and metabolic pathways, the exploration of their specific locations within the digestive tract, and their interactions with other microbes remain to be explored. Such information can contribute to understanding the roles and ecological services provided by symbiotic microbes in the physiology of invasive insects. This information will be crucial for understanding how symbiotic contributions enhance the ability of invasive insects to adapt to new environments and could pave the way for identifying novel microbial targets to effectively manage highly invasive species such as JB.

## Data Availability

The datasets presented in this study can be found in online repositories. The names of the repository/repositories and accession number(s) can be found below: https://www.ncbi.nlm.nih.gov/, PRJNA868936; https://www.mg-rast.org, IDs mgm4872201.3 to mgm4872224.3.

## References

[ref1] Abellan-Schneyder I. Matchado M. S. Reitmeier S. Sommer A. Sewald Z. Baumbach J.. (2021). Primer, pipelines, parameters: issues in 16S rRNA gene sequencing. mSphere 6:e01202-20. doi: 10.1128/msphere.01202-01220 33627512 PMC8544895

[ref2] Althoff E. R. Rice K. B. (2022). Japanese beetle (Coleoptera: Scarabaeidae) invasion of North America: history, ecology, and management. J. Integr. Pest Manag. 13:2. doi: 10.1093/jipm/pmab043

[ref3] Avila-Arias H. Scharf M. E. Turco R. F. Richmond D. S. (2022). Soil environments influence gut prokaryotic communities in the larvae of the invasive Japanese beetle *Popillia japonica* Newman. Front. Microbiol. 13:854513. doi: 10.3389/fmicb.2022.854513, PMID: 35572692 PMC9094118

[ref4] Avila-Arias H. Turco R. F. Scharf M. E. Groves R. L. Richmond D. S. (2023). Larvae of an invasive scarab increase greenhouse gas emissions from soils and recruit gut mycobiota involved in C and N transformations. Front. Microbiol. 14:1102523. doi: 10.3389/fmicb.2023.1102523 37025631 PMC10072269

[ref5] Baker B. J. De Anda V. Seitz K. W. Dombrowski N. Santoro A. E. Lloyd K. G. (2020). Diversity, ecology and evolution of Archaea. Nat. Microbiol. 5, 887–900. doi: 10.1038/s41564-020-0715-z, PMID: 32367054

[ref6] Bolyen E. Rideout J. R. Dillon M. R. Bokulich N. A. Abnet C. C. Al-Ghalith G. A.. (2019). Reproducible, interactive, scalable and extensible microbiome data science using QIIME 2. Nat. Biotechnol. 37, 852–857. doi: 10.1038/s41587-019-0209-9, PMID: 31341288 PMC7015180

[ref7] Borrel G. Brugère J.-F. Gribaldo S. Schmitz R. A. Moissl-Eichinger C. (2020). The host-associated archaeome. Nat. Rev. Microbiol. 18, 622–636. doi: 10.1038/s41579-020-0407-y, PMID: 32690877

[ref8] Brauman A. Doré J. Eggleton P. Bignell D. Breznak J. A. Kane M. D. (2001). Molecular phylogenetic profiling of prokaryotic communities in guts of termites with different feeding habits. FEMS Microbiol. Ecol. 35, 27–36. doi: 10.1111/j.1574-6941.2001.tb00785.x, PMID: 11248387

[ref9] Brune A. (2019). “Methanogenesis in the digestive tracts of insects and other arthropods” in Biogenesis of Hydrocarbons. eds. Stams A. J. M. Sousa D. Z. (Cham: Springer International Publishing), 229–260.

[ref10] Bueno De Mesquita C. P. Wu D. Tringe S. G. (2023). Methyl-based methanogenesis: an ecological and genomic review. Microbiol. Mol. Biol. Rev. 87:e0002422. doi: 10.1128/mmbr.00024-22, PMID: 36692297 PMC10029344

[ref11] Cazemier A. E. Verdoes J. C. Reubsaet F. A. Hackstein J. H. Van Der Drift C. Op Den Camp H. J. (2003). *Promicromonospora pachnodae* sp. nov., a member of the (hemi)cellulolytic hindgut flora of larvae of the scarab beetle *Pachnoda marginata*. Antonie Van Leeuwenhoek 83, 135–148. doi: 10.1023/A:1023325817663, PMID: 12785307

[ref12] Chong J. Liu P. Zhou G. Xia J. (2020). Using MicrobiomeAnalyst for comprehensive statistical, functional, and meta-analysis of microbiome data. Nat. Protoc. 15, 799–821. doi: 10.1038/s41596-019-0264-1, PMID: 31942082

[ref13] Chouaia B. Goda N. Mazza G. Alali S. Florian F. Gionechetti F.. (2019). Developmental stages and gut microenvironments influence gut microbiota dynamics in the invasive beetle *Popillia japonica* Newman (Coleoptera: Scarabaeidae). Environ. Microbiol. 21, 4343–4359. doi: 10.1111/1462-2920.14797, PMID: 31502415

[ref14] Cragg S. M. Beckham G. T. Bruce N. C. Bugg T. D. H. Distel D. L. Dupree P.. (2015). Lignocellulose degradation mechanisms across the Tree of Life. Curr. Opin. Chem. Biol. 29, 108–119. doi: 10.1016/j.cbpa.2015.10.018, PMID: 26583519 PMC7571853

[ref15] Dai Y.-R. Reed D. W. Millstein J. H. Hartzell P. L. Grahame D. A. Demoll E. (1998). Acetyl-CoA decarbonylase/synthase complex from *Archaeoglobus fulgidus*. Arch. Microbiol. 169, 525–529. doi: 10.1007/s002030050606, PMID: 9575239

[ref16] D'alò F. Zucconi L. Onofri S. Canini F. Cannone N. Malfasi F.. (2023). Effects of 5-year experimental warming in the Alpine belt on soil Archaea: Multi-omics approaches and prospects. Environ. Microbiol. Rep. 15, 291–297. doi: 10.1111/1758-2229.13152, PMID: 36999249 PMC10316362

[ref17] Deans C. Krischik V. (2023). The current state and future potential of microbial control of scarab pests. Appl. Sci. 13:766. doi: 10.3390/app13020766

[ref18] Della Rocca F. Milanesi P. (2022a). The new dominator of the world: modeling the global distribution of the Japanese beetle under land use and climate change scenarios. Land 11:567. doi: 10.3390/land11040567

[ref19] Della Rocca F. Milanesi P. (2022b). The spread of the Japanese beetle in a European human-dominated landscape: high anthropization favors colonization of *Popillia japonica*. Diversity 14:658. doi: 10.3390/d14080658

[ref20] Dhariwal A. Chong J. Habib S. King I. L. Agellon L. B. Xia J. (2017). MicrobiomeAnalyst: a web-based tool for comprehensive statistical, visual and meta-analysis of microbiome data. Nucleic Acids Res. 45, W180–W188. doi: 10.1093/nar/gkx295, PMID: 28449106 PMC5570177

[ref21] Fischer P. Q. Sánchez-Andrea I. Stams A. J. M. Villanueva L. Sousa D. Z. (2021). Anaerobic microbial methanol conversion in marine sediments. Environ. Microbiol. 23, 1348–1362. doi: 10.1111/1462-2920.15434, PMID: 33587796 PMC8048578

[ref22] Frew A. Barnett K. Nielsen U. N. Riegler M. Johnson S. N. (2016). Belowground ecology of scarabs feeding on grass roots: current knowledge and future directions for management in Australasia. Front. Plant Sci. 7:321. doi: 10.3389/fpls.2016.00321, PMID: 27047506 PMC4802167

[ref23] Frias J. Garriga A. Peñalver Á. Teixeira M. Beltrí R. Toubarro D.. (2023). Exploring Gut Microbiome Variations between *Popillia japonica* Populations of Azores. Microorganisms 11:1972. doi: 10.3390/microorganisms11081972, PMID: 37630532 PMC10459852

[ref24] Gaci N. Borrel G. Tottey W. O'toole P. W. Brugère J. F. (2014). Archaea and the human gut: new beginning of an old story. World J. Gastroenterol. 20, 16062–16078. doi: 10.3748/wjg.v20.i43.16062, PMID: 25473158 PMC4239492

[ref25] Gan H. Liang C. Wickings K. (2018). Root herbivores accelerate carbon inputs to soil and drive changes in biogeochemical processes. Rhizosphere 6, 112–115. doi: 10.1016/j.rhisph.2018.06.003

[ref26] Gan H. Wickings K. (2020). Root herbivory and soil carbon cycling: Shedding “green” light onto a “brown” world. Soil Biol. Biochem. 150:107972. doi: 10.1016/j.soilbio.2020.107972

[ref27] Görres C.-M. Kammann C. (2020). First field estimation of greenhouse gas release from European soil-dwelling Scarabaeidae larvae targeting the genus *Melolontha*. PLoS One 15:e0238057. doi: 10.1371/journal.pone.0238057, PMID: 32845917 PMC7449402

[ref28] Grahame D. A. Gencic S. Demoll E. (2005). A single operon-encoded form of the acetyl-CoA decarbonylase/synthase multienzyme complex responsible for synthesis and cleavage of acetyl-CoA in *Methanosarcina thermophila*. Arch. Microbiol. 184, 32–40. doi: 10.1007/s00203-005-0006-3, PMID: 16044263

[ref29] Gurung K. Wertheim B. Falcao Salles J. (2019). The microbiome of pest insects: it is not just bacteria. Entomol. Exp. Appl. 167, 156–170. doi: 10.1111/eea.12768

[ref30] Haber M. Burgsdorf I. Handley K. M. Rubin-Blum M. Steindler L. (2021). Genomic insights into the lifestyles of thaumarchaeota inside sponges. Front. Microbiol. 11:622824. doi: 10.3389/fmicb.2020.622824, PMID: 33537022 PMC7848895

[ref31] Hackstein J. H. Stumm C. K. (1994). Methane production in terrestrial arthropods. Proc. Natl. Acad. Sci. USA 91, 5441–5445. doi: 10.1073/pnas.91.12.5441, PMID: 8202505 PMC44011

[ref32] Hackstein J. H. P. Van Alen T. A. (2018). “Methanogens in the Gastrointestinal Tract of Animals” in Microbiology Monographs. ed. Hackstein J. H. P.. 2nd ed (Cham: Springer International Publishing), 121–152.

[ref33] Huang S.-W. Zhang H.-Y. Marshall S. Jackson T. A. (2010). The scarab gut: a potential bioreactor for bio-fuel production. Insect Sci. 17, 175–183. doi: 10.1111/j.1744-7917.2010.01320.x

[ref34] Jansson J. K. Hofmockel K. S. (2018). The soil microbiome—from metagenomics to metaphenomics. Curr. Opin. Microbiol. 43, 162–168. doi: 10.1016/j.mib.2018.01.013, PMID: 29454931

[ref35] Jiménez D. J. Chaves-Moreno D. Van Elsas J. D. (2015). Unveiling the metabolic potential of two soil-derived microbial consortia selected on wheat straw. Sci. Rep. 5:13845. doi: 10.1038/srep13845, PMID: 26343383 PMC4561380

[ref36] Johnson S. N. Rasmann S. (2015). Root-feeding insects and their interactions with organisms in the rhizosphere. Annu. Rev. Entomol. 60, 517–535. doi: 10.1146/annurev-ento-010814-020608, PMID: 25564744

[ref37] Kammann C. Ratering S. Görres C.-M. Guillet C. Müller C. (2017). Stimulation of methane oxidation by CH_4_-emitting rose chafer larvae in well-aerated grassland soil. Biol. Fertil. Soils 53, 491–499. doi: 10.1007/s00374-017-1199-8

[ref38] Kanehisa M. Sato Y. Kawashima M. Furumichi M. Tanabe M. (2016). KEGG as a reference resource for gene and protein annotation. Nucleic Acids Res. 44, D457–D462. doi: 10.1093/nar/gkv1070, PMID: 26476454 PMC4702792

[ref39] Kay M. Wobbrock J. (2019). “ARTool: aligned rank transform for nonparametric factorial ANOVAs” in R package version 0.10.6.

[ref40] Keegan K. P. Glass E. M. Meyer F. (2016). “MG-RAST, a Metagenomics Service for Analysis of Microbial Community Structure and Function” in Microbial Environmental Genomics (MEG). eds. Martin F. Uroz S. (New York, NY: Springer New York), 207–233. 10.1007/978-1-4939-3369-3_13 26791506

[ref41] Keegan K. P. Trimble W. L. Wilkening J. Wilke A. Harrison T. D'souza M.. (2012). A platform-independent method for detecting errors in metagenomic sequencing data: DRISEE. PLoS Comput. Biol. 8:e1002541. doi: 10.1371/journal.pcbi.1002541, PMID: 22685393 PMC3369934

[ref42] Kent W. J. (2002). BLAT--the BLAST-like alignment tool. Genome Res. 12, 656–664. doi: 10.1101/gr.229202, PMID: 11932250 PMC187518

[ref43] Kim J. Y. Whon T. W. Mi Young L. Kim Y. B. Kim N. Min-Sung K.. (2020). The human gut archaeome: identification of diverse haloarchaea in Korean subjects. Microbiome 8, 1–17. doi: 10.1186/s40168-020-00894-x 32753050 PMC7409454

[ref44] Kistner-Thomas E. J. (2019). The potential global distribution and voltinism of the Japanese Beetle (Coleoptera: Scarabaeidae) under current and future climates. J. Insect Sci. 19, 1–13. doi: 10.1093/jisesa/iez023, PMID: 30900722 PMC6429693

[ref45] Klammsteiner T. Walter A. Bogataj T. Heussler C. D. Stres B. Steiner F. M.. (2020). The core gut microbiome of Black soldier fly (*Hermetia illucens*) larvae raised on low-bioburden diets. Front. Microbiol. 11:993. doi: 10.3389/fmicb.2020.00993, PMID: 32508795 PMC7253588

[ref46] Langmead B. Salzberg S. L. (2012). Fast gapped-read alignment with Bowtie 2. Nat. Methods 9, 357–359. doi: 10.1038/nmeth.1923, PMID: 22388286 PMC3322381

[ref47] Lemke T. Stingl U. Egert M. Friedrich M. W. Brune A. (2003). Physicochemical conditions and microbial activities in the highly alkaline gut of the humus-feeding larva of *Pachnoda ephippiata* (Coleoptera: Scarabaeidae). Appl. Environ. Microbiol. 69, 6650–6658. doi: 10.1128/AEM.69.11.6650-6658.2003, PMID: 14602625 PMC262302

[ref48] Love M. I. Huber W. Anders S. (2014). Moderated estimation of fold change and dispersion for RNA-seq data with DESeq2. Genome Biol. 15:550. doi: 10.1186/s13059-014-0550-8, PMID: 25516281 PMC4302049

[ref49] Lyu Z. Liu Y. (2019). “Diversity and Taxonomy of Methanogens” in Biogenesis of Hydrocarbons. eds. Stams A. J. M. Sousa D. Z. (Cham: Springer International Publishing), 19–77.

[ref50] Macleod G. R. Richmond D. S. Filley T. R. (2024). Invasive Japanese beetle (*Popillia japonica* Newman) larvae alter structure and carbon distribution in infested surface soil. Sci. Total Environ. 918:170687. doi: 10.1016/j.scitotenv.2024.170687, PMID: 38320711

[ref51] Majeed M. Z. Miambi E. Barois I. Randriamanantsoa R. Blanchart E. Brauman A. (2014). Contribution of white grubs (Scarabaeidae: Coleoptera) to N_2_O emissions from tropical soils. Soil Biol. Biochem. 75, 37–44. doi: 10.1016/j.soilbio.2014.03.025

[ref52] Martino C. Morton J. T. Marotz C. A. Thompson L. R. Tripathi A. Knight R.. (2019). A novel sparse compositional technique reveals microbial perturbations. mSystems 4, e00016–e00019. doi: 10.1128/msystems.00016-19, PMID: 30801021 PMC6372836

[ref53] Mcgenity T. J. Sorokin D. Y. (2018). “Methanogens and methanogenesis in hypersaline environments” in Biogenesis of Hydrocarbons, Handbook of Hydrocarbon and Lipid Microbiology. eds. Stams A. J. M. Sousa D. Z. (Berlin: Springer International Publishing AG), 665–680.

[ref54] Meyer F. Paarmann D. D'souza M. Olson R. Glass E. M. Kubal M.. (2008). The metagenomics RAST server – a public resource for the automatic phylogenetic and functional analysis of metagenomes. BMC Bioinformatics 9:386. doi: 10.1186/1471-2105-9-386, PMID: 18803844 PMC2563014

[ref55] O’Leary N. A. Wright M. W. Brister J. R. Ciufo S. Haddad D. Mcveigh R.. (2015). Reference sequence (RefSeq) database at NCBI: current status, taxonomic expansion, and functional annotation. Nucleic Acids Res. 44, D733–D745. doi: 10.1093/nar/gkv1189, PMID: 26553804 PMC4702849

[ref56] Oren A. (2014). “The Family Methanosarcinaceae” in The Prokaryotes: Other Major Lineages of Bacteria and The Archaea. eds. Rosenberg E. Delong E. F. Lory S. Stackebrandt E. Thompson F. (Berlin, Heidelberg: Springer Berlin Heidelberg), 259–281.

[ref57] Palkova L. Tomova A. Repiska G. Babinska K. Bokor B. Mikula I.. (2021). Evaluation of 16S rRNA primer sets for characterisation of microbiota in paediatric patients with autism spectrum disorder. Sci. Rep. 11:6781. doi: 10.1038/s41598-021-86378-w, PMID: 33762692 PMC7991656

[ref58] Poggi S. Desneux N. Jactel H. Tayeh C. Verheggen F. (2022). A nationwide pest risk analysis in the context of the ongoing Japanese beetle invasion in continental Europe: the case of metropolitan France. Front. Insect Sci. 2:1079756. doi: 10.3389/finsc.2022.1079756, PMID: 38468800 PMC10926453

[ref59] Potter D. A. Held D. W. (2002). Biology and management of the Japanese beetle. Annu. Rev. Entomol. 47, 175–205. doi: 10.1146/annurev.ento.47.091201.145153, PMID: 11729073

[ref60] Prasad R. K. Chatterjee S. Sharma S. Mazumder P. B. Vairale M. G. Raju P. S. (2018). “Insect Gut Bacteria and Their Potential Application in Degradation of Lignocellulosic Biomass: A Review” in Bioremediation: Applications for Environmental Protection and Management. eds. Varjani S. J. Agarwal A. K. Gnansounou E. Gurunathan B. (Singapore: Springer Singapore), 277–299.

[ref61] Protasov E. Nonoh J. O. Kästle Silva J. M. Mies U. S. Hervé V. Dietrich C.. (2023). Diversity and taxonomic revision of methanogens and other archaea in the intestinal tract of terrestrial arthropods. Front. Microbiol. 14:1281628. doi: 10.3389/fmicb.2023.1281628, PMID: 38033561 PMC10684969

[ref62] Protasov E. Reeh H. Liu P. Poehlein A. Platt K. Heimerl T.. (2024). Genome reduction in novel, obligately methyl-reducing Methanosarcinales isolated from arthropod guts (Methanolapillus gen. nov. and Methanimicrococcus). FEMS Microbiol. Ecol. 100:fiae111. doi: 10.1093/femsec/fiae111, PMID: 39108084 PMC11362671

[ref63] Pruitt K. D. Tatusova T. Maglott D. R. (2007). NCBI reference sequences (RefSeq): a curated non-redundant sequence database of genomes, transcripts and proteins. Nucleic Acids Res. 35, D61–D65. doi: 10.1093/nar/gkl842, PMID: 17130148 PMC1716718

[ref64] Purdy K. J. (2007). “The distribution and diversity of Euryarchaeota in termite guts” in Advances in applied microbiology (Amsterdam: Elsevier Press) 62, 63–80. 17869602 10.1016/S0065-2164(07)62003-6

[ref65] Richmond D. S. (2022). Managing white grubs in turfgrass. Available online at: https://extension.entm.purdue.edu/publications/E-271/E-271.html.

[ref66] Sage R. F. (2020). Global change biology: A primer. Glob. Chang. Biol. 26, 3–30. doi: 10.1111/gcb.14893, PMID: 31663217

[ref67] Scharf M. E. Peterson B. F. (2021). A century of synergy in termite symbiosis research: Linking the past with new genomic insights. Annu. Rev. Entomol. 66, 23–43. doi: 10.1146/annurev-ento-022420-074746, PMID: 33417825

[ref68] Shakya M. Quince C. Campbell J. H. Yang Z. K. Schadt C. W. Podar M. (2013). Comparative metagenomic and rRNA microbial diversity characterization using archaeal and bacterial synthetic communities. Environ. Microbiol. 15, 1882–1899. doi: 10.1111/1462-2920.12086, PMID: 23387867 PMC3665634

[ref69] Shanovich H. N. Dean A. N. Koch R. L. Hodgson E. W. (2019). Biology and management of Japanese beetle (Coleoptera: Scarabaeidae) in corn and soybean. J. Integr. Pest Manag. 10, 1–14. doi: 10.1093/jipm/pmz009

[ref70] Somee M. R. Amoozegar M. A. Dastgheib S. M. M. Shavandi M. Maman L. G. Bertilsson S.. (2022). Genome-resolved analyses show an extensive diversification in key aerobic hydrocarbon-degrading enzymes across bacteria and archaea. BMC Genomics 23:690. doi: 10.1186/s12864-022-08906-w, PMID: 36203131 PMC9535955

[ref71] Starke R. Siles J. A. Fernandes M. L. P. Schallert K. Benndorf D. Plaza C.. (2021). The structure and function of soil archaea across biomes. J. Proteome 237:104147. doi: 10.1016/j.jprot.2021.104147, PMID: 33582288

[ref72] Swingle M. C. (1931). The influence of soil acidity on the pH value of the contents of the digestive tract of Japanese beetle larvae. Ann. Entomol. Soc. Am. 24, 496–502.

[ref73] Thijs S. Op De Beeck M. Beckers B. Truyens S. Stevens V. Van Hamme J. D.. (2017). Comparative evaluation of four bacteria-specific primer pairs for 16S rRNA gene surveys. Front. Microbiol. 8:494. doi: 10.3389/fmicb.2017.00494, PMID: 28400755 PMC5368227

[ref74] Thomas C. M. Desmond-Le Quéméner E. Gribaldo S. Borrel G. (2022). Factors shaping the abundance and diversity of the gut archaeome across the animal kingdom. Nat. Commun. 13:3358. doi: 10.1038/s41467-022-31038-4, PMID: 35688919 PMC9187648

[ref75] Treonis A. M. Grayston S. J. Murray P. J. Dawson L. A. (2005). Effects of root feeding, cranefly larvae on soil microorganisms and the composition of rhizosphere solutions collected from grassland plants. Appl. Soil Ecol. 28, 203–215. doi: 10.1016/j.apsoil.2004.08.004

[ref76] Vázquez-Baeza Y. Pirrung M. Gonzalez A. Knight R. (2013). EMPeror: a tool for visualizing high-throughput microbial community data. GigaScience 2, 1–4. doi: 10.1186/2047-217X-2-16, PMID: 24280061 PMC4076506

[ref77] Wada N. Iwabuchi N. Sunairi M. Nakajima M. Iwata R. Anzai H. (2020). Site-specific profiles of biochemical properties in the larval digestive tract of Japanese rhinoceros beetle, *Trypoxylus dichotomus* (Coleoptera: Scarabaeidae). Entomol. Sci. 23, 33–43. doi: 10.1111/ens.12394

[ref78] Weiss S. Xu Z. Z. Peddada S. Amir A. Bittinger K. Gonzalez A.. (2017). Normalization and microbial differential abundance strategies depend upon data characteristics. Microbiome 5:27. doi: 10.1186/s40168-017-0237-y, PMID: 28253908 PMC5335496

[ref79] Wilke A. Harrison T. Wilkening J. Field D. Glass E. M. Kyrpides N.. (2012). The M5nr: a novel non-redundant database containing protein sequences and annotations from multiple sources and associated tools. BMC Bioinformatics 13:141. doi: 10.1186/1471-2105-13-141, PMID: 22720753 PMC3410781

[ref80] Wobbrock J. O. Findlater L. Gergle D. Higgins J. J. (2011). The aligned rank transform for nonparametric factorial analyses using only anova procedures. In: Proceedings of the SIGCHI Conference on Human Factors in Computing Systems. Vancouver, BC: Association for Computing Machinery.

[ref81] Zhou Y. Staver A. C. Davies A. B. (2023). Species-level termite methane production rates. Ecology 104:e3905. doi: 10.1002/ecy.3905, PMID: 36314967

