# Supplementary material for: Metagenomic analysis reveals methanogenic and other archaeal genes in the digestive tract of invasive Japanese beetle larvae and associated soil
Source: Front Microbiol. 2025 Jul 25;16:1609893. doi: 10.3389/fmicb.2025.1609893 (PMC12332493; doi:10.3389/fmicb.2025.1609893)
Supplement: Supplementary file 1 [file Supplementary_file_1.zip › Figure S1.pdf]

Supplementary Figures

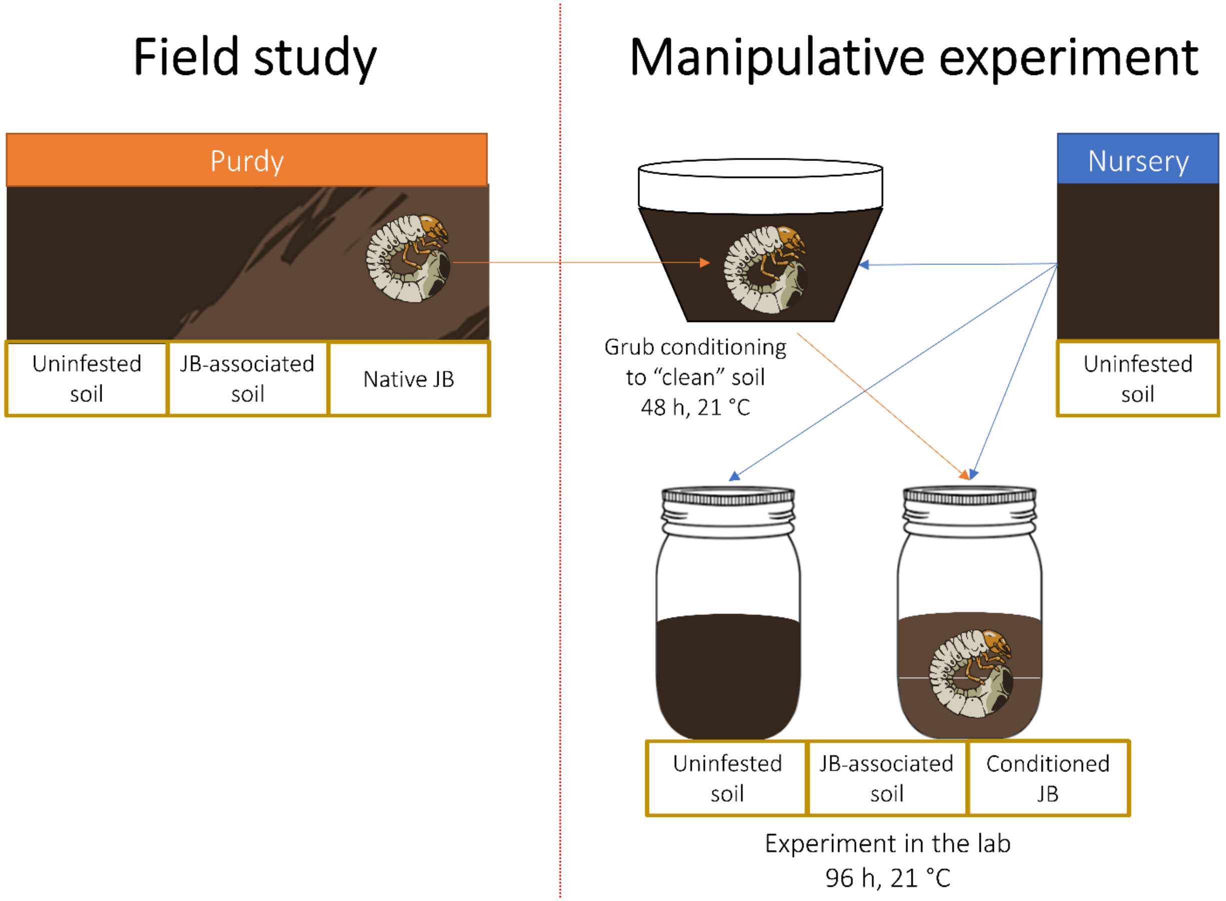

Figure S1. Experimental design to examine how the gut archaeome of third instar larvae of the Japanese beetle *Popillia japonica* Newman (JB) collected from a naturally JB-infested location (Purdy Sod Farm, Lafayette, IN, USA) would be altered by incubating those larvae in soil taken from a JB-free location (Purdue University Nursery, West Lafayette, IN, USA), and to determine how the soil archaeome is impacted by JB infestation. Two JBs per grub sample OR two soil DNA extractions per soil sample were used. A total of 4 sets of samples from each study (i.e., field study, manipulative laboratory experiment) were rendered, as follows: JB midgut, JB hindgut, JB infested soil, and uninfested soil. Each set was performed by triplicate for a total of 24 samples.
